# Using the P-CaRES Tool to Identify Palliative Care Needs in Patients with Life-Limiting Diseases: An Analysis of Internal Medicine Admissions

**DOI:** 10.3390/jcm14124206

**Published:** 2025-06-13

**Authors:** Luise Fidelsberger, Claudia Fischer, Gudrun Kreye, Eleonora Meran, Rudolf Likar, Raphael van Tulder, Haro Stettner, Eva Katharina Masel, Josef Singer, Nguyen-Son Le

**Affiliations:** 1 Division of Palliative Care, Division of Internal Medicine 2, University Hospital Krems, 3500 Krems, Austria; 2 Karl Landsteiner University of Health Sciences, 3500 Krems, Austria; 3 Department of Health Economics, Center for Public Health, Medical University of Vienna, 1090 Vienna, Austria; 4 Department of Internal Medicine 2, Hospital of St. John of God, 1020 Vienna, Austria; 5 Department of Anaesthesiology and Intensive Care, Medicine, Klinikum Klagenfurt am Wörthersee, 9020 Klagenfurt, Austria; 6 Division of Internal Medicine 1, University Hospital Krems, 3500 Krems, Austria; 7 Institute for Statistics, Alpen-Adria University Klagenfurt, 9020 Klagenfurt, Austria; 8 Department of Internal Medicine I, Clinical Division of Palliative Medicine, Medical University Vienna, 1090 Vienna, Austria

**Keywords:** palliative care, screening tool, palliative care needs, life-limiting disease, P-CaRES tool, timely referral

## Abstract

**Background/Objectives:** Early integration of palliative care (PC) improves outcomes for patients with life-limiting diseases (LLDs). This study evaluated the effectiveness of the Palliative Care and Rapid Emergency Screening (P-CaRES) tool—originally developed for emergency settings—in identifying unmet PC needs among patients admitted to internal medicine wards. **Methods:** In this retrospective study, the P-CaRES tool was applied to medical records of patients with LLDs. Demographic and clinical data were extracted from charts. Logistic regression identified predictors of PC receipt; survival was analyzed using Kaplan–Meier estimates and log-rank tests. **Results:** Among 2509 patients screened, 631 (23.9%) had at least one LLD. Of these, 451 (71.5%) were identified as having PC needs. However, only 132 (20.9%) received PC services—126 with documented need and 6 without. Advanced cancer (OR = 6.46, *p* < 0.001), a positive response to the surprise question (OR = 4.88, *p* = 0.008), and frequent hospitalizations (OR = 2.24, *p* < 0.001) predicted PC receipt. Median survival declined with increasing disease burden (10 vs. 372 days for patients with ≥3 vs. 1 LLD), unmet PC needs (85 vs. 1383 days), and a “yes” response to the surprise question (79 vs. 1598 days) (all *p* < 0.001). **Conclusions:** The P-CaRES tool effectively identified PC needs in patients with LLDs, including those with cancer. Clinical indicators such as frequent hospital admissions, a positive response to the surprise question, and multimorbidity predicted both the need for PC and shorter survival. Nonetheless, substantial gaps existed between identified needs and PC delivery—especially for non-cancer patients. Structured screening and timely referrals may bridge this gap and improve care for seriously ill individuals.

## 1. Introduction

As the population ages, the number of people living with at least one life-limiting disease (LLD) is growing rapidly [1]. Patients with multiple chronic conditions are frequently admitted to internal medicine departments, creating complex challenges for healthcare professionals [2]. These challenges include managing multifaceted symptom profiles, navigating emotionally and ethically demanding situations, and communicating about prognosis and care goals with patients and families [3,4]. Persistent misconceptions about palliative care (PC), limited knowledge among clinicians, and constrained resources often hinder the timely identification and support of patients with unmet needs [5,6]. Recognizing the limits of curative treatment and considering the wishes of both patients and their families are essential to delivering the best possible care for multimorbid patients [7].

The integration of PC alongside standard medical treatment is essential when curative options are no longer effective [8]. This is underlined by the World Health Organization (WHO), which emphasizes that PC should be provided early in the course of the disease, in conjunction with other life-prolonging therapies [9]. PC aims to provide comprehensive support for patients by relieving uncontrolled symptoms such as pain, breathlessness, and fatigue; addressing psychological, social, and spiritual distress; and supporting complex decision-making and advanced care planning. It also ensures coordinated care across settings and essential support for caregivers, thereby improving quality of life for both patients and their families [9,10]. Without timely PC, patients are at risk of unmanaged symptoms, increased psychological distress, and poor communication about care preferences, often resulting in longer hospital stays, higher healthcare costs, and reduced quality of life [11,12,13,14].

Despite the well-documented benefits of timely PC, many patients who could benefit from PC remain unidentified [11,12,13,14,15,16,17,18,19,20]. Contributing factors include limited knowledge among clinicians and persistent misunderstandings regarding the scope and purpose of PC [21]. To ensure timely care, effective and standardized screening methods are needed to detect unmet needs [22]. Studies have demonstrated that a large proportion of patients with LLDs in hospital settings have significant, yet poorly identified, unmet PC needs, leading to unmanaged symptoms and less favorable outcomes [23,24]. A systematic review by El Mokhallalati et al. identified 25 studies reporting the use or development of screening tools to identify patients with likely unmet PC needs [25]. While these tools showed significant variability in accuracy (sensitivity 3–94%, specificity 26–99%), their evaluation was limited due to a lack of a valid comparator, leaving their true clinical utility uncertain. The review emphasized the need for standardized screening processes that not only predict mortality and deterioration but also anticipate comprehensive PC needs and the rate of functional decline [25]. Addressing gaps in the identification and delivery of PC can improve symptom control, resource use, and patient-centered outcomes. Early and systematic screening enables holistic care and timely referrals, empowering healthcare teams to provide care that aligns with individual needs and values [11,12,14].

The Palliative Care and Rapid Emergency Screening (P-CaRES) tool addresses these challenges by providing a structured, practical, and easy-to-use instrument that helps non-PC specialists identify patients with LLDs who may benefit from PC. Originally developed for emergency settings, P-CaRES is intended to support timely referrals, promote interdisciplinary collaboration, and enhance patient-centered care [26]. Validated as a reliable instrument [27,28,29,30,31], P-CaRES is, so far, the only screening tool adapted for Primary Palliative Care for Emergency Medicine (PRIM-ER) intervention [29]. Additionally, P-CaRES has been validated by PC experts and used in several studies across different settings [27,28,32,33,34,35,36], including an Austrian ED setting, where it was translated into German [28]. A systematic review by Kirkland et al. analyzed 35 studies on 14 screening tools for unmet PC needs in EDs [30]. P-CaRES was the second most frequently studied, featured in eight studies, and was noted for its role in identifying PC needs in EDs. While the widely known “Surprise Question” (SQ) showed moderate sensitivity and specificity in predicting mortality, the review highlighted the P-CaRES tool as valuable for identifying unmet PC needs, though further research was advised for broad implementation [30].

Regarding screening tools to identify patients with LLDs in internal medicine departments, literature is sparse. As patients with LLDs are mostly found at internal medicine departments, we aimed to determine whether the P-CaRES tool was feasible to screen for patients with LLDs and PC needs in an inpatient setting in internal medicine departments.

## 2. Materials and Methods

### 2.1. Study Population

A retrospective chart review was conducted at the University Hospital in Krems, Austria, across two internal medicine departments: the Department of Internal Medicine 1 (cardiology) and the Department of Internal Medicine 2 (hemato-oncology, gastroenterology, and palliative care).

Patients aged 18 years and older admitted during two selected time periods—1 October to 31 December 2019 (pre-coronavirus disease (COVID-19)), and 1 March to 31 May 2020 (during COVID-19)—were included in the study, provided that complete medical records were available. The break between these periods reflects the local transition phase at the onset of the COVID-19 pandemic, during which hospital processes and patient flows were rapidly adapting. The selected timeframes allowed for a comprehensive assessment of patient characteristics and care needs under varying circumstances. Patients with incomplete data were excluded, as were those admitted during both time periods, to avoid potential confounding effects from readmissions that could skew the analysis, particularly regarding outcomes that may not have accurately reflected initial treatment.

### 2.2. Data Collection

The data collection was performed using the records available at the University Hospital of Krems via the access-limited computer system. Patients were pseudonymized using sequential numbers. Any access to patient records was personalized and monitored. Study-relevant data were pseudonymously compiled and evaluated. Only authorized people within the research team had access to the original data.

### 2.3. The P-CaRES Screening Tool

**Figure 1:**
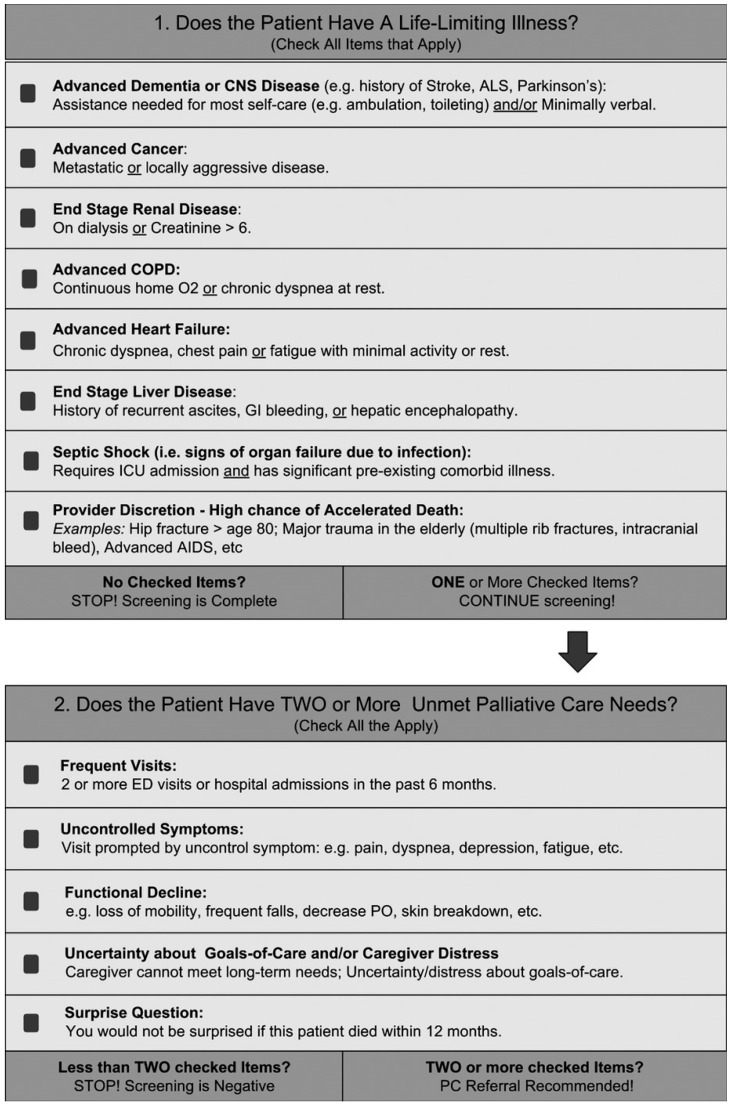
The P-CaRES screening tool [26].

The P-CaRES tool [26] was retrospectively applied to the extracted clinical records by the research team to systematically identify patients with LLDs and PC needs. (Figure 1). We used an adapted German version [28] of the 2-tier screening tool originally developed for EDs for the identification of patients with PC needs to screen for patients with LLDs and PC needs at internal medicine departments.

The P-CaRES tool screening procedure consists of two parts (Figure 1). Part 1 assesses whether patients have LLDs. According to the P-CaRES tool, conditions that classify as LLDs include advanced dementia, advanced cancer, end-stage renal disease, advanced chronic obstructive pulmonary disease (COPD), advanced heart failure, end-stage liver disease, and septic shock with signs of organ failure due to infection. The P-CaRES tool offers specific criteria to define disease progression as “life-limiting”; for instance, patients with end-stage renal disease must either require dialysis or have a serum creatinine level >6 mg/dL to meet the LLD criteria.

Additionally, a ‘provider discretion’ category allows medical professionals to classify patients with a high likelihood of accelerated death due to other conditions not covered in the listed categories. This flexibility ensures that nuanced clinical observations, such as trajectory and complex comorbidities, are accounted for, as described in George’s original study [26].

If no LLD is identified, the screening concludes. If one or more LLDs are present, the screening proceeds to Part 2, which examines unmet PC needs. Unmet PC needs are indicated by factors such as frequent hospital or ED visits within the past 6 months, uncontrolled symptoms, functional decline, uncertainty about goals of care, caregiver distress, or a positive response to the SQ (i.e., if it would not be surprising if the patient died within 12 months). If two or more of these criteria are met, the P-CaRES tool recommends a PC referral [26].

### 2.4. The Specialized PC Service at the University-Based Referral Center

At the University Hospital Krems, specialized PC is a service provided by PC specialists who coordinate comprehensive and interdisciplinary care for patients. Our multidisciplinary team includes physicians, nurses, psychologists, and other allied health professionals, all of whom have completed recognized specialist training and have substantial experience in the field. This distinguishes our SPC service from general PC, which can be provided by non-specialist healthcare professionals as part of routine care. At our university hospital, all patients identified as having PC needs who are referred to the SPC team by the attending physicians are co-managed by our SPC team, ensuring that those with complex or advanced needs receive comprehensive, expert-led care and support.

### 2.5. Statistical Analysis

Descriptive statistics were used to summarize the patient cohort. The primary endpoint was the prevalence of PC integration; the secondary endpoint was overall survival (OS), defined as the time from first admission to death from any cause or last follow-up. Age distributions were compared using the Kruskal–Wallis test, and gender using the chi-square test.

Group comparisons (e.g., PC needs vs. PC received, patients with LLDs vs. subgroups) were conducted using appropriate tests based on data type and distribution:

- Categorical variables: chi-square or Fisher’s exact test;
- Non-normally distributed continuous variables: Mann–Whitney U test (pairwise) and Kruskal–Wallis test (multiple groups), with post-hoc analyses as needed;
- Normality: assessed using the Shapiro–Wilk test.

Boxplots were created to graphically represent the distributions of age and length of hospital stay. OS was determined retrospectively based on hospital records and administrative follow-up data, independent of the P-CaRES tool. Survival probabilities were estimated using the Kaplan–Meier method, and differences between survival curves were assessed with the log-rank test. Patients alive at the time of analysis or lost to follow-up were censored at the date of last contact.

Univariate and multivariate logistic regression analyses were performed to identify predictors of PC referral and assess whether patients identified by the P-CaRES tool as needing PC actually received it.

All *p*-values were from two-sided tests, with a significance level of ≤0.05. Analyses were conducted using SPSS version 28 (Statistical Package for the Social Sciences Inc., Armonk, NY, USA).

## 3. Results

### 3.1. Clinical Characteristics of Patients

#### 3.1.1. Patient Screening and Study Population

**Figure 2:**
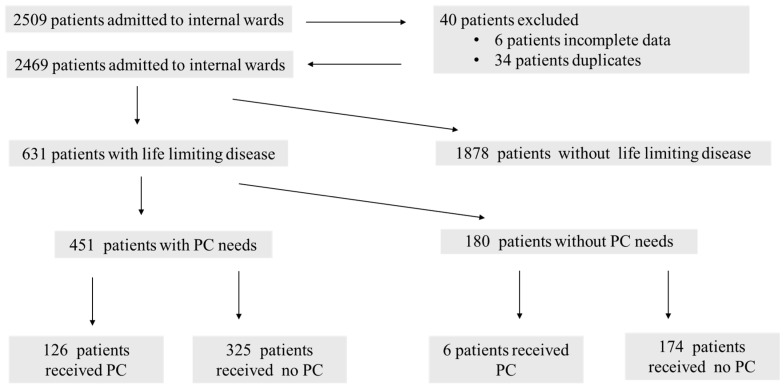
Flowchart of enrolled patients. Abbreviation: PC = palliative care.

A total of 2509 patients were screened for LLDs and PC needs during the two study periods (Figure 2). Of these, 6 patients were excluded due to incomplete data, and 34 patients who were admitted in both time periods were removed to avoid duplication. Of the remaining 2469 patients, 1878 patients (76.1%) had no LLD and were therefore not included in the final analysis (Figure 2). The remaining 631 patients (23.9%) had at least one LLD and formed the cohort analyzed in detail.

#### 3.1.2. Identification and Receipt of Palliative Care

Among these 631 patients with at least one LLD, 451 (71.5%) were identified as having PC needs based on the two-tier screening process. PC was provided to 132 patients (20.9%), of whom 126 (95.5%) had an indication for PC. Notably, six patients (1.0%) received PC despite not meeting the indication criteria (Table 1).

#### 3.1.3. Demographics and Diagnoses

The median age of the cohort was 78.0 years, with an interquartile range (IQR) of 18.0 years, indicating that 50% of the patients were between 69 and 87 years old. A total of 57.8% were male. Patients with PC needs were slightly older (median 80.0 years, IQR 16.0). The most common LLDs were advanced cancer (30.9%), neurological diseases or dementia (20.8%), and COPD (13.9%). Among those who received PC with an indication, advanced cancer (59.5%), neurological disease/dementia (11.9%), COPD (7.9%), and cardiac diseases (7.9%) were the most prevalent conditions.

#### 3.1.4. Palliative Care Indicators

Provider discretion of a high chance for accelerated death was documented in 34.1% of all patients and in 41.9% of those with PC needs. Other common indicators among patients with PC needs included frequent visits to doctors or hospitals (46.8%), uncontrolled symptoms (92.9%), and functional decline (45.9%). The SQ was answered “no” for 69.1% of all patients and 91.1% of those with PC needs.

#### 3.1.5. Healthcare Utilization and Outcomes

The median length of hospital stay was 6.0 days (IQR 9.0) overall and slightly longer among patients who received PC (7.0 days, IQR 12.0). The subgroup who received PC without an indication had the shortest stay (2.5 days, IQR 4.0) but the highest number of hospital admissions (3.0, IQR 1.5).

#### 3.1.6. Statistical Differences Across Patient Groups

Age distribution differed significantly across the groups (*p* = 0.009). Pairwise comparisons revealed that patients with PC needs (median 80.0 years) were significantly older than those with LLDs without PC needs (median 72.5 years, *p* = 0.006) and those who received PC (median 77.5 years, *p* = 0.015, Figure S1). Gender distribution, with a slightly higher proportion of males in each category, showed no significant differences.

Among the primary diagnoses, advanced cancer was significantly more common in the group that received PC (59.5%) compared with those with PC needs (28.6%, *p* < 0.001. In contrast, neurological disease or dementia was more frequently observed in the PC needs group (24.2%) than in those who received PC (11.9%, *p* = 0.003). Further details can be found in Table 2 and Table 3.

Patients with advanced cancer and PC needs were most likely to receive PC (58.1%), followed by those with liver disease (29.4%) and hematological malignancies (25.8%). In contrast, patients with septic shock with signs of organ failure and PC needs had the lowest proportion receiving PC (10.7%), followed by those with COPD (11.4%) and neurological diseases (13.8%). Overall, the likelihood of receiving PC was significantly higher for patients with advanced cancer compared with those with non-cancer diagnoses (58.1% vs. 17.4%, *p* < 0.001).

#### 3.1.7. Differences in Clinical Indicators Across Patient Groups

Among clinical characteristics, uncontrolled symptoms were significantly more common in patients with PC needs (92.9%) than in the overall LLD cohort (79.9%) (*p* < 0.001). However, no significant differences were observed between the PC needs group and the PC-received group (92.9%, *p* = 0.985).

Although frequent hospital visits were descriptively more common in patients who received PC (53.2%) compared with those with PC needs (46.8%), this difference did not reach statistical significance (*p* = 0.204). Further details can be found in Table 2 and Table 3.

### 3.2. Multivariate Predictors of Receiving Palliative Care

In the multivariate logistic regression model (binary outcome) restricted to patients with identified PC needs, several factors were significantly associated with the likelihood of receiving PC.

Patients with an advanced cancer diagnosis had over six times higher odds of receiving PC compared to those without (OR = 6.46, *p* < 0.001). Similarly, patients for whom the SQ was answered positively were nearly five times more likely to receive PC (OR = 4.88, *p* = 0.008).

A higher number of hospital admissions was also associated with increased likelihood of receiving PC (OR = 2.24, *p* < 0.001). In contrast, patients with neurological disease or dementia were less likely to receive PC (OR = 0.63), but this association did not reach statistical significance (*p* = 0.17). Provider-based discretion for an accelerated death was not independently associated with receiving PC when controlling for other factors (*p* = 0.92).

### 3.3. Overall Survival Analyses

#### 3.3.1. Overall Survival Analysis for Patients with LLDs (n = 631) Related to the Number of LLDs

**Figure 3:**
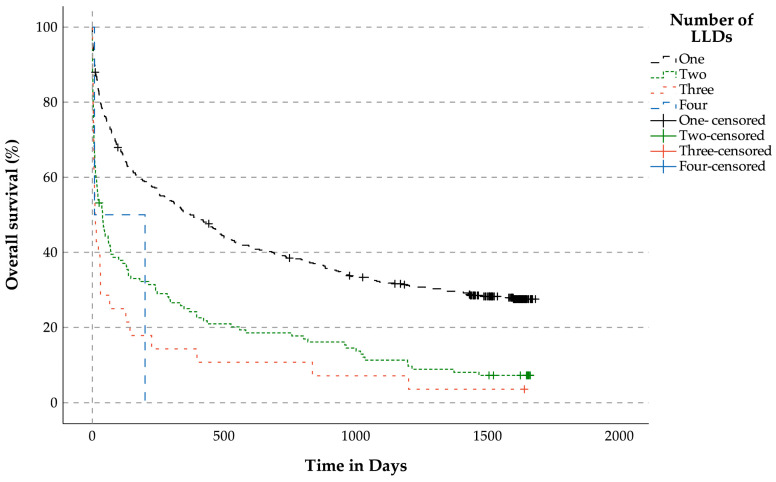
Overall survival analysis for patients with life-limiting diseases (n = 631) related to the number of life-limiting diseases.

Probability estimates of median OS were as follows: 372 days for patients with one LLD, 39 days for patients with two LLDs, 10 days for patients with three LLDs days, and 8 days for patients with four LLDs (*p* < 0.001, Figure 3).

#### 3.3.2. OS Related to the Prevalence of Unmet Palliative Care Needs for Patients with LLDs

**Figure 4:**
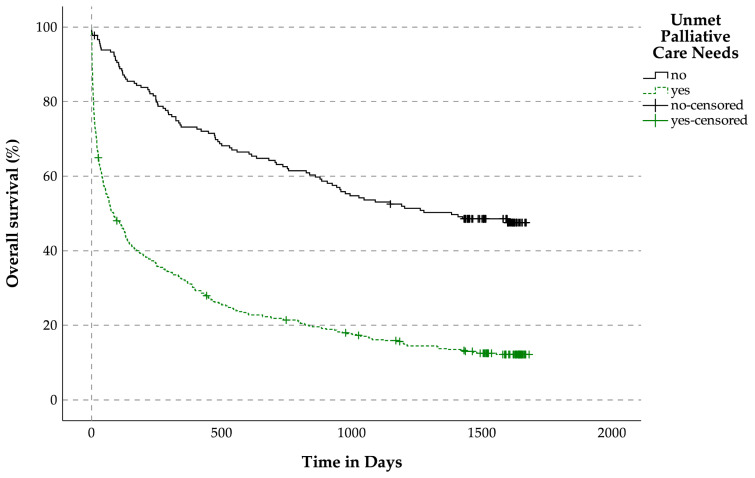
Overall survival related to the prevalence of unmet palliative care needs for patients with life-limiting diseases.

The median OS for patients without unmet PC needs was 1383 days, while those with unmet PC needs had a median OS of 85 days, (*p* < 0.001, Figure 4).

#### 3.3.3. OS Related to the Prevalence of Palliative Care Services for Patients with LLDs

**Figure 5:**
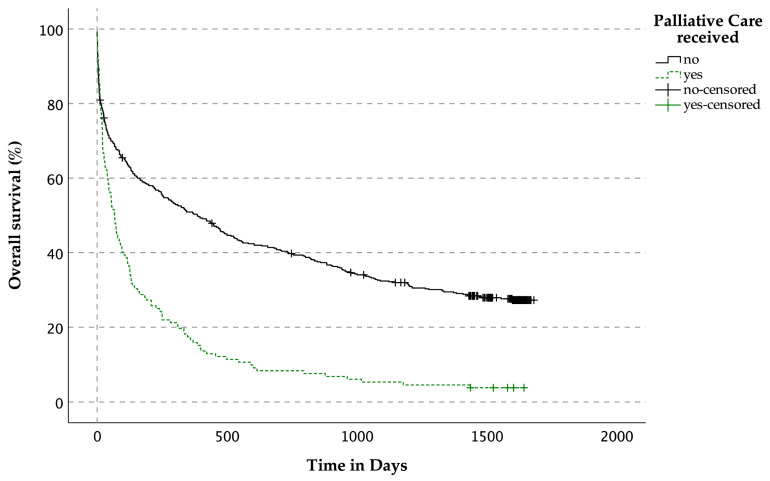
Overall survival related to the prevalence of palliative care services for patients with life-limiting diseases.

The median OS for patients not receiving PC services was 385 days, while those patients receiving PC services had a median OS of 67 days, (*p* < 0.001, Figure 5).

#### 3.3.4. OS Related to Indication for Palliative Care Services and Reception of Palliative Care Services for Patients with LLDs

**Figure 6:**
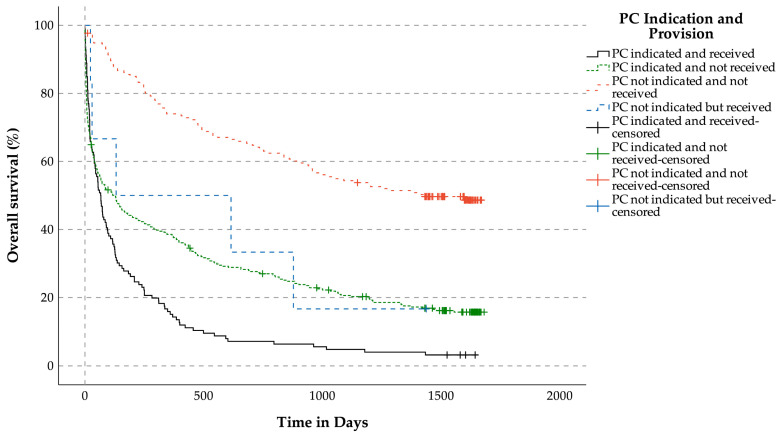
Overall survival related to indication for palliative care services and provision of palliative care services for patients with life-limiting diseases. Abbreviation: PC = palliative care.

Median OS for patients without indication for PC services and not receiving PC services was 1426 days. Median OS for patients without indication for PC services but receiving PC services was 131 days. Median OS for patients with indication for PC services but who did not get PC services was 123 days. Median OS for patients with indication for PC services who received PC services was 66 days (*p* < 0.001, Figure 6).

#### 3.3.5. OS Related to the Surprise Question for Patients with LLDs (Figure 7)

The median OS for patients whose clinicians answered ‘yes’ to the SQ was 79 days, while those whose clinicians answered ‘no’ had a median OS of 1598 days, (*p* < 0.001).

## 4. Discussion

This study confirms the high prevalence of PC needs among patients with LLDs admitted to internal medicine wards and highlights substantial gaps between identified needs and the provision of PC services. Using the P-CaRES screening tool in combination with clinical indicators, we were able to identify patients with significant unmet needs and demonstrate key disparities in care delivery. The application of P-CaRES in this setting, beyond its initial validation in EDs, underscores its potential utility for broader hospital-based screening strategies [12].

Among the 631 patients with LLDs, more than 70% were identified as having PC needs, yet only one in five received specialist PC services. These results mirror findings from prior studies conducted in EDs and outpatient settings, where validated tools have shown effectiveness in identifying PC needs but have not always translated into increased referrals due to workflow barriers and clinical uncertainty [27,30].

While patients with advanced cancer were more likely to receive specialist PC, those with non-malignant LLDs were underrepresented among those referred—despite being identified as having PC needs. This reflects a persistent trend in clinical practice and a known diagnostic bias, where non-malignant conditions are often under-recognized in terms of PC eligibility [27,37,38]. The use of structured screening tools like P-CaRES may help standardize the identification of PC needs and promote more equitable access, independent of diagnosis.

Our multivariate analysis identified several significant predictors of receiving PC among patients with PC needs. Advanced cancer diagnosis, a positive answer to the SQ (surprise question), and a higher number of hospital admissions were independently associated with a higher likelihood of receiving PC services. These findings align with earlier work demonstrating the prognostic value of the SQ and its utility in identifying patients at elevated risk of mortality [30,39].

Survival analyses confirmed the prognostic relevance of both multimorbidity and PC-related factors. Patients with multiple LLDs had progressively shorter median OS (overall survival). Similarly, patients with unmet PC needs had significantly shorter survival, likely due to uncontrolled symptoms, psychological distress, and inadequate support—all of which contribute to higher hospitalization and mortality rates [40,41]. Additionally, frailty, multimorbidity, and lack of social support further exacerbate patient vulnerability, while communication barriers within healthcare systems often delay appropriate interventions [42]. Interestingly, our findings revealed that patients with PC had shorter OS. This likely reflects the prevailing practice of late PC referrals, often initiated when patients already exhibit poor performance status and advanced symptoms—factors that independently predict reduced survival [43]. Indeed, studies indicate that patients referred for PC within the last 30 days of life have worse outcomes compared to those referred earlier, highlighting the need for earlier identification and proactive integration of PC to maximize patient benefit [12,14,26,43].

The analysis of OS based on PC indication and actual service provision further underscores the importance of timely identification and referral. Patients without a PC indication who did not receive PC lived substantially longer than those in any other group, reflecting earlier stages of illness and lower care needs. In contrast, patients with a documented need for PC had significantly shorter survival, regardless of whether they ultimately received PC services. Notably, those who received PC despite no formal indication also had limited survival, suggesting that clinical judgment may have identified unmet needs not captured by the screening tool. Together, these findings highlight both the prognostic relevance of needs-based screening and the critical timing of referral processes in internal medicine settings, as patients referred for PC often have only limited time remaining [12,13,14]. Earlier identification of PC needs has been associated with reduced hospitalizations, shorter length of stay, and lower end-of-life healthcare costs, primarily through increased use of home- and community-based services and reduced acute care utilization. While some studies show overall cost savings, others report a redistribution of resources, but consistently demonstrate improved patient-centered care and decreased aggressive interventions [12,13,14,41]. These findings underscore the relevance of timely PC not only for patient outcomes but also for healthcare system sustainability.

Although the SQ has been criticized for its limited predictive accuracy and lack of evidence regarding clinical and economic outcomes [44], our findings support its value as part of a broader screening strategy. Patients for whom the SQ was answered with ‘no’ had a median OS of only 79 days, compared with 1598 days in patients for whom the answer was ‘yes’. This suggests that, while the SQ should not be used in isolation for clinical decision-making, it can provide meaningful insights into prognosis when combined with structured tools such as P-CaRES. This is in line with findings from Yen et al., who demonstrated that using both the SQ and a formal screening tool significantly improved the accuracy of predicting 12-month mortality compared with either alone [45]. Similarly, while Downar et al. concluded that the SQ performs modestly overall and worse for non-cancer patients, it may still serve as a useful complement to other criteria [46].

The diagnostic bias in PC referral patterns remains a pressing concern. Our data show that patients with advanced cancer were up to six times more likely to receive PC than those with non-cancer diagnoses, even when needs were identified. In contrast, neurological diagnoses were underrepresented among patients who received PC. These disparities are consistent with the literature and highlight a need for diagnosis-neutral approaches to PC referral, guided by need rather than prognosis alone [27,38]. Barriers to PC for non-cancer patients, particularly those with neurological conditions, include prognostic uncertainty, limited provider training, misconceptions about the relevance of PC, and less-established structured referral pathways in non-malignant diseases [47,48,49]. Additionally, communication challenges—especially in patients with cognitive or speech impairments—can further delay recognition of PC needs [50]. Addressing these barriers requires targeted education and the development of diagnosis-neutral referral criteria [51].

From a clinical perspective, our findings reinforce the value of structured screening using tools such as P-CaRES and the SQ to identify patients with unmet PC needs not only in EDs, where the tool was originally developed, but also in inpatient settings. Tools like this offer a feasible, low-cost approach to integrating PC screening into daily practice, particularly when combined with education and referral pathways to ensure actionable outcomes. Experiences from adaptations of NECPAL and other screening tools further suggest that cultural tailoring and workflow integration are essential for successful implementation [52].

A recent study by Schmitz et al. validated the German version of the P-CaRES tool in an ED setting, demonstrating strong agreement with expert assessment and the SPICT (Supportive and Palliative Care Indicators Tool), and confirming its face and content validity within the German healthcare context [31]. Their contribution represents an important step toward integrating structured PC screening into acute care environments. While their focus was on the ED, our study expanded the scope of application by retrospectively evaluating the P-CaRES tool in an inpatient setting—specifically among patients admitted to internal medicine wards.

To our knowledge, this is the first study to investigate the use of P-CaRES in this clinical context. By applying the tool to a larger patient population with LLDs, we were able to explore its practical relevance for identifying unmet PC needs beyond emergency care, highlighting the potential value of structured screening in routine hospital practice. To operationalize P-CaRES in routine internal medicine practice, integration into electronic health records, staff education, and establishment of clear referral pathways are essential. Experiences from other settings show that multidisciplinary collaboration, regular auditing, and embedding screening into admission workflows improve compliance and timely follow-up [12,29]. Institutional support and ongoing training are key to sustainable implementation. Our findings support the development of hospital-wide policies for standardized PC screening, as structured tools like P-CaRES are feasible and effective across various settings, including EDs and intensive care units, where early PC identification remains a challenge [6,28,31]. Implementing standardized PC screening protocols across various hospital departments can facilitate timely referrals, align treatments with patient goals, and optimize resource utilization.

### Limitations

This study had several limitations. First, the single-center design may have limited generalizability, and findings should be validated in multicenter or cross-national settings. Second, the retrospective nature of the study introduced potential for documentation bias, particularly regarding the completeness of PC indicators and clinical judgment. Third, although we statistically accounted for overlap between PC needs and PC receipt, the small size of the “PC received without indication” group limited deeper subgroup analysis. Additionally, survival outcomes may reflect clinical triage processes and are not necessarily causally linked to PC provision. Lastly, certain psychosocial, spiritual, or caregiver-related PC needs may not have been captured in the data, potentially underestimating the true burden of unmet needs.

## 5. Conclusions

Our study demonstrates that systematic screening using the P-CaRES tool in internal medicine wards effectively identifies patients with LLDs and unmet PC needs. While patients with advanced cancer were more likely to receive PC services, those with non-malignant conditions remained underserved. Clinical indicators such as frequent hospital admissions, the surprise question, and multimorbidity were predictive of both PC needs and shorter survival. These findings support broader implementation of standardized screening tools in hospital settings and highlight the urgent need to reduce disparities in PC access by ensuring referral decisions are guided by patient needs rather than diagnostic labels.

## Figures and Tables

**Figure 7:**
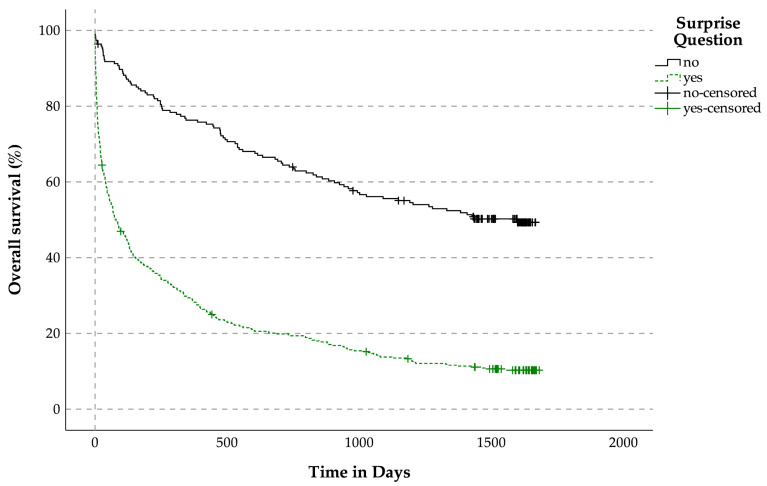
Overall survival related to the surprise question for patients with life-limiting diseases.

**Table 1:** Patient characteristics, palliative care needs, and service utilization.

Characteristic	Patients with Life-Limiting Diseases	Patients with PC Needs	Patients with PC Needs Who Received PC	Patients Who Received PC Without Needs
Total	631 (100%)	451 (71.5%)	126 (20.0%)	6 (1.0%)
Male	365 (57.8%)	262 (58.1%)	72 (57.1%)	3 (50.0%)
Female	266 (42.2%)	189 (41.9%)	54 (42.9%)	3 (50.0%)
Neurological disease/dementia	131 (20.8%)	109 (24.2%)	15 (11.9%)	0 (0.0%)
Advanced cancer	195 (30.9%)	129 (28.6%)	75 (59.5%)	3 (50.0%)
Hematological malignancy	51 (8.1%)	31 (6.9%)	8 (6.3%)	2 (33.3%)
End-stage renal disease	21 (3.3%)	16 (3.5%)	5 (4.0%)	0 (0.0%)
End-stage COPD	88 (13.9%)	59 (13.1%)	10 (7.9%)	0 (0.0%)
End-stage cardiac disease	70 (11.1%)	54 (12.0%)	10 (7.9%)	0 (0.0%)
End-stage liver disease	17 (2.7%)	14 (3.1%)	5 (4.0%)	0 (0.0%)
Septic shock with signs of organ failure	28 (4.4%)	24 (5.3%)	3 (2.4%)	0 (0.0%)
Provider-based identification	215 (34.1%)	189 (41.9%)	36 (28.6%)	1 (16.7%)
Frequent visits	227 (36.0%)	211 (46.8%)	67 (53.2%)	2 (33.3%)
Uncontrolled symptoms	504 (79.9%)	419 (92.9%)	117 (92.9%)	1 (16.7%)
Functional decline	211 (33.4%)	207 (45.9%)	55 (43.7%)	0 (0.0%)
Uncertainty regarding goals of care	98 (15.5%)	98 (21.7%)	24 (19.0%)	0 (0.0%)
Surprise question	436 (69.1%)	411 (91.1%)	122 (96.8%)	3 (50.0%)
Consultation by PC team indicated	451 (71.5%)	451 (100.0%)	126 (100.0%)	0 (0.0%)
Patient consulted by PC team	132 (20.9%)	126 (27.9%)	126 (100.0%)	6 (100.0%)
Age at admission (median)	78.0 years (IQR 18.0)	80.0 years (IQR 16.0)	77.5 years (IQR 16.0)	71.5 years (IQR 15.2)
Length of hospital stay (median)	6.0 days (IQR 9.0)	6.0 days (IQR 10.0)	7.0 days (IQR 12.0)	2.5 days (IQR 4.0)
Number of admissions (median)	1.0 (IQR 1.0)	1.0 (IQR 1.0)	1.0 (IQR 1.0)	3.0 (IQR 1.5)

Abbreviations: COPD = chronic obstructive pulmonary disease, IQR = interquartile range, PC = palliative care. Note: All clinical indicators (including “frequent visits”, “uncontrolled symptoms”, and “functional decline”) are defined according to the P-CaRES tool criteria (See Figure 1). PC should be indicated only in patients with ≥2 PC needs according to 2-tier screening. Six patients received PC despite not fulfilling this criterion.

**Table 2:** Comparison of diagnoses and clinical indicators between patients with palliative care needs and those who received palliative care.

Diagnoses and Clinical Indicators	Patients with PC Needs	Patients with PC Needs Who Received PC	*p* Value
Neurological disease/dementia	109 (24.2%)	15 (11.9%)	0.003
Advanced cancer	129 (28.6%)	75 (59.5%)	<0.001
Hematological malignancy	31 (6.9%)	8 (6.3%)	0.836
End-stage renal disease	16 (3.5%)	5 (4.0%)	0.824
End-stage COPD	59 (13.1%)	10 (7.9%)	0.116
End-stage cardiac disease	54 (12.0%)	10 (7.9%)	0.202
End-stage liver disease	14 (3.1%)	5 (4.0%)	0.631
Septic shock with signs of organ failure	24 (5.3%)	3 (2.4%)	0.167
Provider-based identification	189 (41.9%)	36 (28.6%)	0.007
Frequent visits	211 (46.8%)	67 (53.2%)	0.204
Uncontrolled symptoms	419 (92.9%)	117 (92.9%)	0.985
Functional decline	207 (45.9%)	55 (43.7%)	0.654
Uncertainty regarding goals of care	98 (21.7%)	24 (19.0%)	0.515
Surprise question	411 (91.1%)	122 (96.8%)	0.033

Abbreviations: COPD = chronic obstructive pulmonary disease, PC = palliative care. Note: All clinical indicators (including “frequent visits”, “uncontrolled symptoms”, and “functional decline”) are defined according to the P-CaRES tool criteria (See Figure 1).

**Table 3:** Comparison of diagnoses and clinical indicators between patients with life-limiting diseases and patients with palliative care needs.

Diagnoses and Clinical Indicators	Patients with Life-Limiting Diseases	Patients with PC Needs	*p* Value
Neurological disease/dementia	131 (20.8%)	109 (24.2%)	0.209
Advanced cancer	195 (30.9%)	129 (28.6%)	0.455
Hematological malignancy	51 (8.1%)	31 (6.9%)	0.532
End-stage renal disease	21 (3.3%)	16 (3.5%)	0.979
End-stage COPD	88 (13.9%)	59 (13.1%)	0.750
End-stage cardiac disease	70 (11.1%)	54 (12.0%)	0.725
End-stage liver disease	17 (2.7%)	14 (3.1%)	0.830
Septic shock with signs of organ failure	28 (4.4%)	24 (5.3%)	0.599
Provider-based identification	215 (34.1%)	189 (41.9%)	0.010
Frequent visits	227 (36.0%)	211 (46.8%)	<0.001
Uncontrolled symptoms	504 (79.9%)	419 (92.9%)	<0.001
Functional decline	211 (33.4%)	207 (45.9%)	<0.001
Uncertainty regarding goals of care	98 (15.5%)	98 (21.7%)	0.011
Surprise question	436 (69.1%)	411 (91.1%)	<0.001

Abbreviations: COPD = chronic obstructive pulmonary disease, PC = palliative care. Note: All clinical indicators (including “frequent visits”, “uncontrolled symptoms”, and “functional decline”) are defined according to the P-CaRES tool criteria (See Figure 1).

## Data Availability

The data presented in this study are available on request from the corresponding author. The data are not publicly available.

## Supplementary Materials

The following supporting information can be downloaded at https://www.mdpi.com/article/10.3390/jcm14124206/s1: Figure S1: Age distribution of patients with life-limiting diseases in relation to palliative care needs and provision, Figure S2: Distribution of length of hospital stay (days) in patients with life-limiting diseases in relation to palliative care needs and provision.

## Abbreviations

The following abbreviations are used in this manuscript:

ED	Emergency department
COPD	Chronic obstructive pulmonary disease
IQR	Interquartile range
LLD	Life-limiting disease
P-CaRES	Palliative Care and Rapid Emergency Screening
PC	Palliative care
OR	Odds ratio
OS	Overall survival
SPICT	Supportive and Palliative Care Indicators Tool
SQ	Surprise question
WHO	World Health Organization

## References

[1] Maresova P., Javanmardi E., Barakovic S., Barakovic Husic J., Tomsone S., Krejcar O., Kuca K. (2019). Consequences of chronic diseases and other limitations associated with old age–a scoping review. BMC Public Health.

[2] Sumner J., Ng C.W.T., Teo K.E.L., Peh A.L.T., Lim Y.W. (2024). Co-designing care for multimorbidity: A systematic review. BMC Med..

[3] Alhatim N.A., AlShehery M.Z. (2024). Exploring the Challenges in Palliative Care As Perceived by the Saudi Physicians: A Cross-Sectional Study. Cureus.

[4] Kashmeeri M., Islam A., Banik P.C. (2024). Challenges experienced by health care providers working in both hospital and home-based palliative care units in Dhaka city: A multi-center based cross-sectional study. PLoS ONE.

[5] Sitima G., Galhardo-Branco C., Reis-Pina P. (2024). Equity of access to palliative care: A scoping review. Int. J. Equity Health.

[6] Weissman D.E., Meier D.E. (2011). Identifying patients in need of a palliative care assessment in the hospital setting: A consensus report from the Center to Advance Palliative Care. J. Palliat. Med..

[7] Kuipers S.J., Nieboer A.P., Cramm J.M. (2021). Making care more patient centered; experiences of healthcare professionals and patients with multimorbidity in the primary care setting. BMC Fam. Pract..

[8] Hui D., Bansal S., Strasser F., Morita T., Caraceni A., Davis M., Cherny N., Kaasa S., Currow D., Abernethy A. (2015). Indicators of integration of oncology and palliative care programs: An international consensus. Ann. Oncol..

[9] WHO Definition of Palliative Care. https://www.who.int/news-room/fact-sheets/detail/palliative-care.

[10] Henson L.A., Maddocks M., Evans C., Davidson M., Hicks S., Higginson I.J. (2020). Palliative Care and the Management of Common Distressing Symptoms in Advanced Cancer: Pain, Breathlessness, Nausea and Vomiting, and Fatigue. J. Clin. Oncol..

[11] Kaasa S., Loge J.H., Aapro M., Albreht T., Anderson R., Bruera E., Brunelli C., Caraceni A., Cervantes A., Currow D.C. (2018). Integration of oncology and palliative care: A Lancet Oncology Commission. Lancet Oncol..

[12] Hui D., Heung Y., Bruera E. (2022). Timely Palliative Care: Personalizing the Process of Referral. Cancers.

[13] Bakitas M.A., Tosteson T.D., Li Z., Lyons K.D., Hull J.G., Li Z., Dionne-Odom J.N., Frost J., Dragnev K.H., Hegel M.T. (2015). Early versus delayed initiation of concurrent palliative oncology care: Patient outcomes in the ENABLE III randomized controlled trial. J. Clin. Oncol..

[14] Temel J.S., Greer J.A., Muzikansky A., Gallagher E.R., Admane S., Jackson V.A., Dahlin C.M., Blinderman C.D., Jacobsen J., Pirl W.F. (2010). Early palliative care for patients with metastatic non–small-cell lung cancer. N. Engl. J. Med..

[15] El-Jawahri A., LeBlanc T., VanDusen H., Traeger L., Greer J.A., Pirl W.F., Jackson V.A., Telles J., Rhodes A., Spitzer T.R. (2016). Effect of inpatient palliative care on quality of life 2 weeks after hematopoietic stem cell transplantation: A randomized clinical trial. JAMA.

[16] Evans C.J., Harding R., Higginson I.J. (2013). ‘Best practice’ in developing and evaluating palliative and end-of-life care services: A meta-synthesis of research methods for the MORECare project. Palliat. Med..

[17] Liu Y.-J., Wu L.-P., Wang H., Han Q., Wang S.-N., Zhang J. (2023). The clinical effect evaluation of multidisciplinary collaborative team combined with palliative care model in patients with terminal cancer: A randomised controlled study. BMC Palliat. Care.

[18] Pantilat S.Z., O’Riordan D.L., Dibble S.L., Landefeld C.S. (2010). Hospital-based palliative medicine consultation: A randomized controlled trial. Arch. Intern. Med..

[19] Rabow M.W., Dibble S.L., Pantilat S.Z., McPhee S.J. (2004). The comprehensive care team: A controlled trial of outpatient palliative medicine consultation. Arch. Intern. Med..

[20] Senderovich H., McFadyen K. (2020). Palliative care: Too good to be true?. Rambam Maimonides Med. J..

[21] Flieger S.P., Chui K., Koch-Weser S. (2020). Lack of awareness and common misconceptions about palliative care among adults: Insights from a national survey. J. Gen. Intern. Med..

[22] Xie Z., Ding J., Jiao J., Tang S., Huang C. (2024). Screening instruments for early identification of unmet palliative care needs: A systematic review and meta-analysis. BMJ Support. Palliat. Care.

[23] Tavabie S., Ta Y., Stewart E., Tavabie O., Bowers S., White N., Seton-Jones C., Bass S., Taubert M., Berglund A. (2024). Seeking Excellence in End of Life Care UK (SEECare UK): A UK multi-centred service evaluation. BMJ Support. Palliat. Care.

[24] Gardiner C., Gott M., Ingleton C., Seymour J., Cobb M., Noble B., Bennett M., Ryan T. (2013). Extent of palliative care need in the acute hospital setting: A survey of two acute hospitals in the UK. Palliat. Med..

[25] ElMokhallalati Y., Bradley S.H., Chapman E., Ziegler L., Murtagh F.E., Johnson M.J., Bennett M.I. (2020). Identification of patients with potential palliative care needs: A systematic review of screening tools in primary care. Palliat. Med..

[26] George N., Barrett N., McPeake L., Goett R., Anderson K., Baird J. (2015). Content Validation of a Novel Screening Tool to Identify Emergency Department Patients With Significant Palliative Care Needs. Acad. Emerg. Med. Off. J. Soc. Acad. Emerg. Med..

[27] Bowman J., George N., Barrett N., Anderson K., Dove-Maguire K., Baird J. (2016). Acceptability and reliability of a novel palliative care screening tool among emergency department providers. Acad. Emerg. Med..

[28] Kostenberger M., Neuwersch S., Weixler D., Pipam W., Zink M., Likar R. (2019). Prevalence of palliative care patients in emergency departments. Wien. Klin. Wochenschr..

[29] Tan A., Durbin M., Chung F.R., Rubin A.L., Cuthel A.M., McQuilkin J.A., Modrek A.S., Jamin C., Gavin N., Mann D. (2020). Design and implementation of a clinical decision support tool for primary palliative Care for Emergency Medicine (PRIM-ER). BMC Med. Inform. Decis. Mak..

[30] Kirkland S.W., Garrido Clua M., Kruhlak M., Villa-Roel C., Couperthwaite S., Yang E.H., Elwi A., O’Neill B., Duggan S., Brisebois A. (2021). Comparison of characteristics and management of emergency department presentations between patients with met and unmet palliative care needs. PLoS ONE.

[31] Schmitz J., Tewes M., Mathew B., Bubel M., Kill C., Risse J., Huessler E.-M., Kowall B., Salvador Comino M.R. (2025). Validation of the Palliative Care and Rapid Emergency Screening (P-CaRES) Tool in Germany. J. Clin. Med..

[32] Ouchi K., Block S.D., Schonberg M.A., Jamieson E.S., Aaronson E.L., Pallin D.J., Tulsky J.A., Schuur J.D. (2017). Feasibility testing of an emergency department screening tool to identify older adults appropriate for palliative care consultation. J. Palliat. Med..

[33] Duffy J., Crump S., O’Connor E. (2019). P034: Identifying unmet palliative care needs in the emergency department. Can. J. Emerg. Med..

[34] Yang C., Yang T.-T., Tsou Y.-J., Lin M.-H., Fan J.-S., Huang H.-H., Tsai M.-C., Yen D.H.-T. (2020). Initiating palliative care consultation for acute critically ill patients in the emergency department intensive care unit. J. Chin. Med. Assoc..

[35] Paske J.R.T., DeWitt S., Hicks R., Semmens S., Vaughan L. (2021). Palliative care and rapid emergency screening tool and the palliative performance scale to predict survival of older adults admitted to the hospital from the emergency department. Am. J. Hosp. Palliat. Med.®.

[36] Tolia V., Yourman L., Brennan J., Cronin A., Castillo E. (2020). 182 Initial Evaluation of a Palliative Care Screening Tool in the Emergency Department. Ann. Emerg. Med..

[37] Gómez-Batiste X., Martínez-Muñoz M., Blay C., Amblàs J., Vila L., Costa X., Espaulella J., Espinosa J., Constante C., Mitchell G.K. (2014). Prevalence and characteristics of patients with advanced chronic conditions in need of palliative care in the general population: A cross-sectional study. Palliat. Med..

[38] Gómez-Batiste X., Turrillas P., Tebé C., Calsina-Berna A., Amblàs-Novellas J. (2022). NECPAL tool prognostication in advanced chronic illness: A rapid review and expert consensus. BMJ Support. Palliat. Care.

[39] Bowers S.P., Black P., McCheyne L., Wilson D., Penfold R.S., Stapleton L., Channer P., Mills S.E., Williams L., Quirk F. (2025). Descriptions of advanced multimorbidity: A scoping review with content analysis. J. Multimorb. Comorbidity.

[40] Bakitas M., Lyons K.D., Hegel M.T., Balan S., Brokaw F.C., Seville J., Hull J.G., Li Z., Tosteson T.D., Byock I.R. (2009). Effects of a palliative care intervention on clinical outcomes in patients with advanced cancer: The Project ENABLE II randomized controlled trial. JAMA.

[41] Kavalieratos D., Corbelli J., Zhang D., Dionne-Odom J.N., Ernecoff N.C., Hanmer J., Hoydich Z.P., Ikejiani D.Z., Klein-Fedyshin M., Zimmermann C. (2016). Association Between Palliative Care and Patient and Caregiver Outcomes: A Systematic Review and Meta-analysis. JAMA.

[42] Murtagh F.E., Bausewein C., Verne J., Groeneveld E.I., Kaloki Y.E., Higginson I.J. (2014). How many people need palliative care? A study developing and comparing methods for population-based estimates. Palliat. Med..

[43] Allard P., Dionne A., Potvin D. (1995). Factors associated with length of survival among 1081 terminally ill cancer patients. J. Palliat. Care.

[44] Davis M.P., Vanenkevort E. (2022). The surprise question. BMJ Support. Palliat. Care.

[45] Yen Y.-F., Lee Y.-L., Hu H.-Y., Sun W.-J., Ko M.-C., Chen C.-C., Wong W.K., Morisky D.E., Huang S.-J., Chu D. (2022). Early palliative care: The surprise question and the palliative care screening tool—Better together. BMJ Support. Palliat. Care.

[46] Downar J., Goldman R., Pinto R., Englesakis M., Adhikari N.K. (2017). The “surprise question” for predicting death in seriously ill patients: A systematic review and meta-analysis. Cmaj.

[47] Gebresillassie B.M., Attia J.R., Mersha A.G., Harris M.L. (2024). Prognostic models and factors identifying end-of-life in non-cancer chronic diseases: A systematic review. BMJ Support. Palliat. Care.

[48] Mounsey L., Ferres M., Eastman P. (2018). Palliative care for the patient without cancer. Aust. J. Gen. Pract..

[49] Hepgul N., Gao W., Evans C.J., Jackson D., van Vliet L.M., Byrne A., Crosby V., Groves K.E., Lindsay F., Higginson I.J. (2018). Integrating palliative care into neurology services: What do the professionals say?. BMJ Support. Palliat. Care.

[50] Zehm A., Hazeltine A.M., Greer J.A., Traeger L., Nelson-Lowe M., Brizzi K., Jacobsen J. (2020). Neurology clinicians’ views on palliative care communication: “How do you frame this?”. Neurol. Clin. Pract..

[51] Hamano J., Shima Y., Kizawa Y. (2023). Current situation and support need for non-cancer patients’ admission to inpatient hospices/palliative care units in Japan: A nationwide multicenter survey. Ann. Palliat. Med..

[52] Lüthi F.T., MacDonald I., Amoussou J.R., Bernard M., Borasio G.D., Ramelet A.-S. (2022). Instruments for the identification of patients in need of palliative care in the hospital setting: A systematic review of measurement properties. JBI Evid. Synth..

