# Supplementary material for: Using the P-CaRES Tool to Identify Palliative Care Needs in Patients with Life-Limiting Diseases: An Analysis of Internal Medicine Admissions
Source: J Clin Med. 2025 Jun 13;14(12):4206. doi: 10.3390/jcm14124206 (PMC12193812; doi:10.3390/jcm14124206)
Supplement: Supplementary file 1 [file jcm-14-04206-s001.zip › jcm-3652693-supplementary.pdf]

Journal of Clinical Medicine

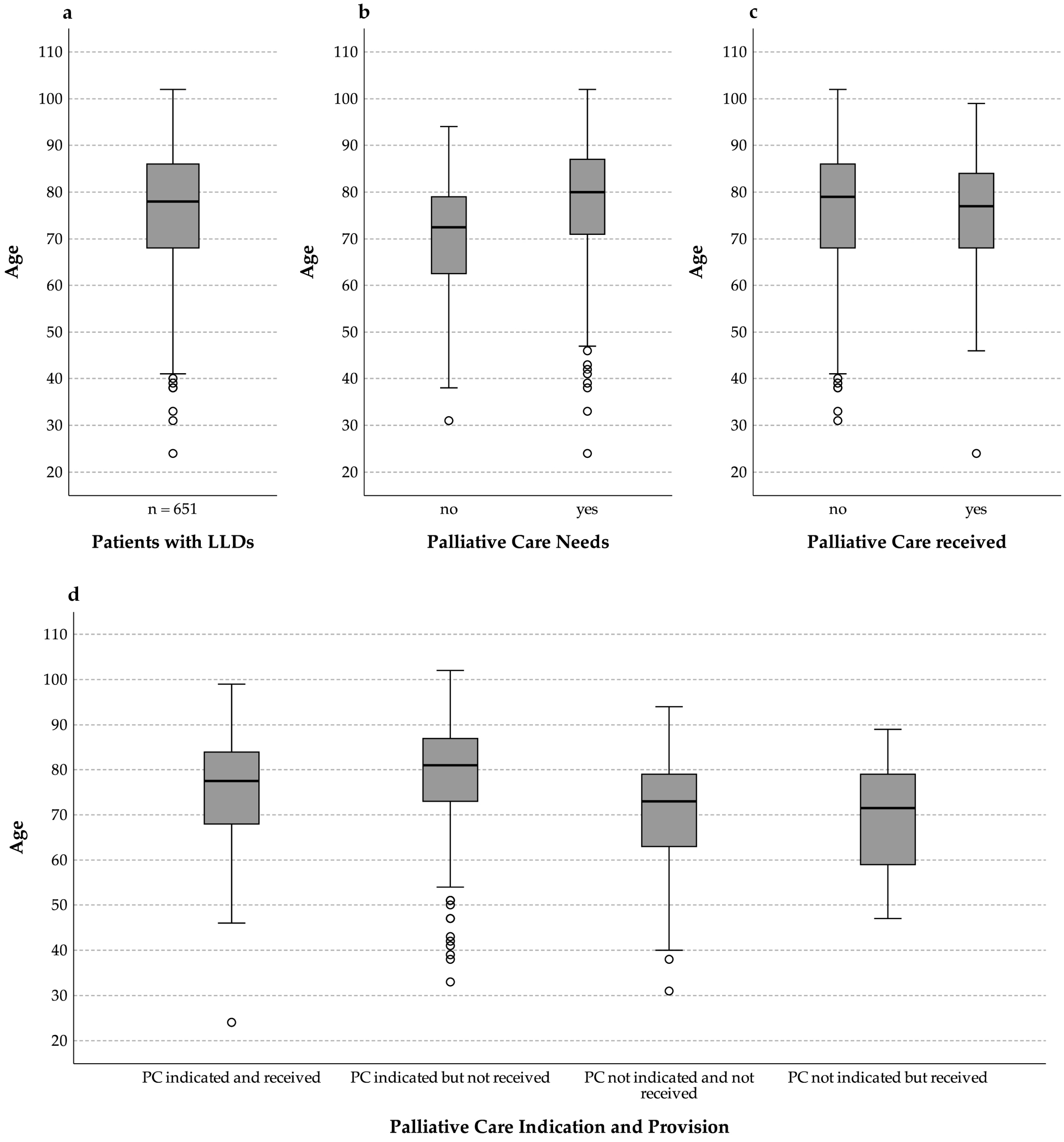

Figure S1. Age distribution of patients with life-limiting diseases in relation to palliative care needs and provision. Abbreviation: PC = palliative care.

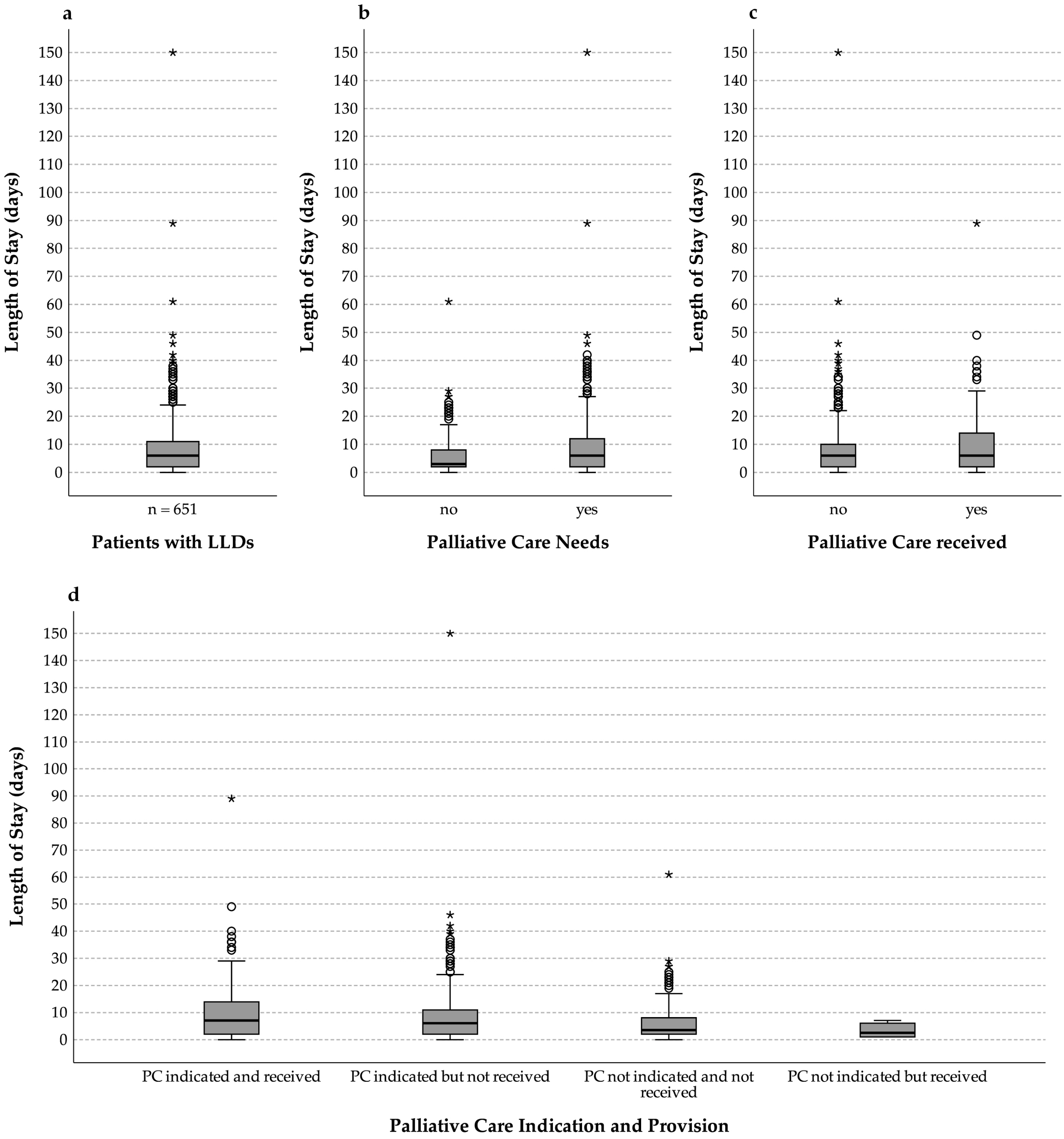

Figure S2. Distribution of length of hospital stay (days) in patients with life-limiting diseases in relation to palliative care needs and provision. Abbreviation: PC = palliative care.

Disclaimer/Publisher's Note: The statements, opinions and data contained in all publications are solely those of the individual author(s) and contributor(s) and not of MDPI and/or the editor(s). MDPI and/or the editor(s) disclaim responsibility for any injury to people or property resulting from any ideas, methods, instructions or products referred to in the content.
